# Supplementary material for: Reproducible Tract Profiles 2 (RTP2) suite, from diffusion MRI acquisition to clinical practice and research
Source: Sci Rep. 2023 Apr 12;13:6010. doi: 10.1038/s41598-023-32924-7 (PMC10097625; doi:10.1038/s41598-023-32924-7)
Supplement: Supplementary file 1 — Supplementary Figures. [file 41598_2023_32924_MOESM1_ESM.pdf]

SUPPLEMENTARY MATERIALS for:

Reproducible Tract Profiles 2 (RTP2): from diffusion MRI acquisition to clinical practice and research

Short Title: Reproducible Tract Profiles

Authors: Garikoitz Lerma-Usabiaga[*1,2,3], Mengxing Liu[2], Pedro M. Paz-Alonso[2,4], Brian A. Wandell[1,3]

Affiliation:
[1] Department of Psychology, Stanford University, 450 Serra Mall, Jordan Hall Building, 94305 Stanford, California, USA
[2] BCBL. Basque Center on Cognition, Brain and Language. Mikeletegi Pasealekua 69, Donostia - San Sebastián, 20009 Gipuzkoa, Spain
[3] Wu Tsai Neurosciences Institute, Stanford University, 94305 Stanford, California, USA
[4] IKERBASQUE. Basque foundation for science. 48013 Bilbao, Spain.

Correspondence:
Garikoitz Lerma-Usabiaga
Email: garikoitz@gmail.com
BCBL. Basque Center on Cognition, Brain and Language.
Mikeletegi Pasealekua 69
Donostia - San Sebastián, 20009 Gipuzkoa, Spain

ORCID:
G.L-U.: 0000-0001-9800-4816; **M.L.:** 0000-0001-8313-137X;
P.M.P-A.: 0000-0002-0325-9304; **B.A.W.:** 0000-0002-2974-1836

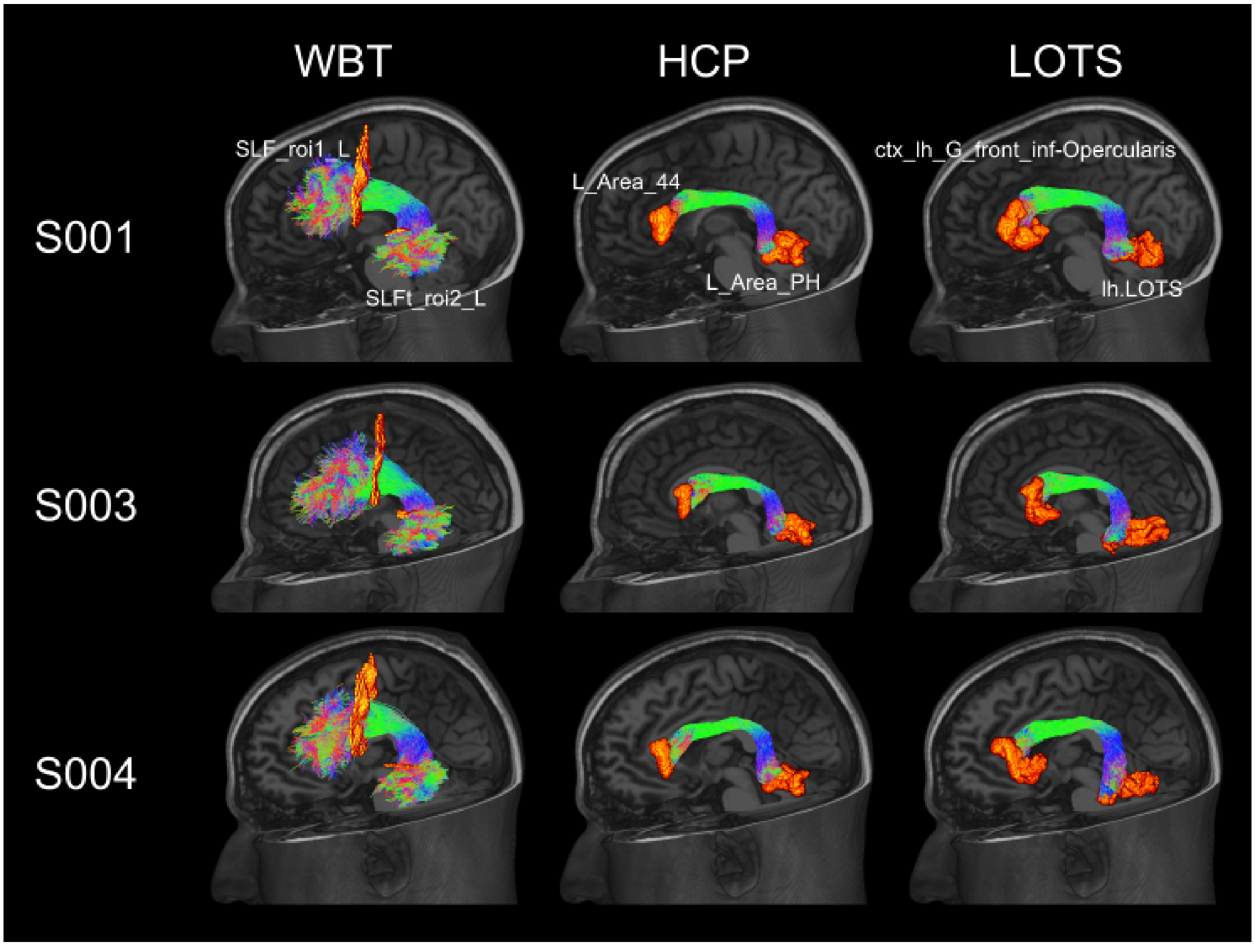

Figure S1. Rendered image of left arcuate fasciculus obtained with three different procedures in three representative brains.
In the WBT procedure, the left AF is obtained by selecting streamlines from the whole-brain-tractogram passing through white matter ROIs. In the HCP procedure, the left AF is obtained by directly tracing between area 44 and area PH from the HCP atlas. In the LOTS procedure, similar to the HCP procedure, the left AF is obtained by directly tracing between two cortical ROIs, opercularis obtained from Freesurfer's cortical atlas, and LOTS manually drawn in Freesurfer's fsaverage surface template. All ROIs are represented in orange, and all fibers are represented in colors attending their direction.

In addition to FA, we measured the reproducibility of tract profiles using other microstructural tractometric indices: axial diffusivity (AD), mean diffusivity (MD) and radial diffusivity (RD). Similar to the reproducibility obtained for FA values, all three indices showed high computational (Supplementary Figure S2) and test-retest reproducibility (Supplementary Figure S3) across all tracts of interest. In test-retest reproducibility, as expected, the correlation coefficients tended to be numerically lower relative to computational reproducibility, but they still show high reproducibility at the group level.

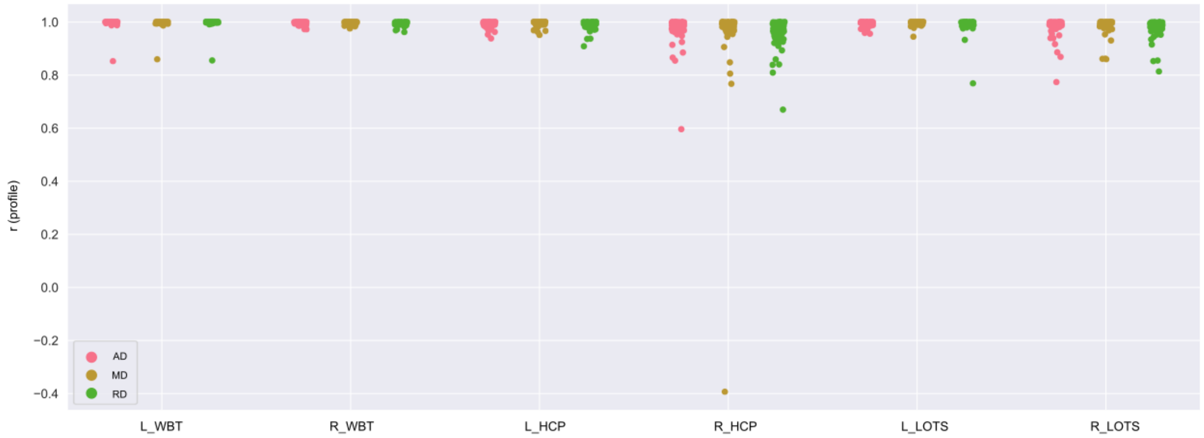

Figure S2. Computational reproducibility of fiber profiles for several microstructural measurements
Correlation coefficients of fiber profiles on axial diffusivity (AD), mean diffusivity (MD) and radial diffusivity (RD) between two separate computations. Each dot represents one instance of the correlation coefficient of one participant between computations.

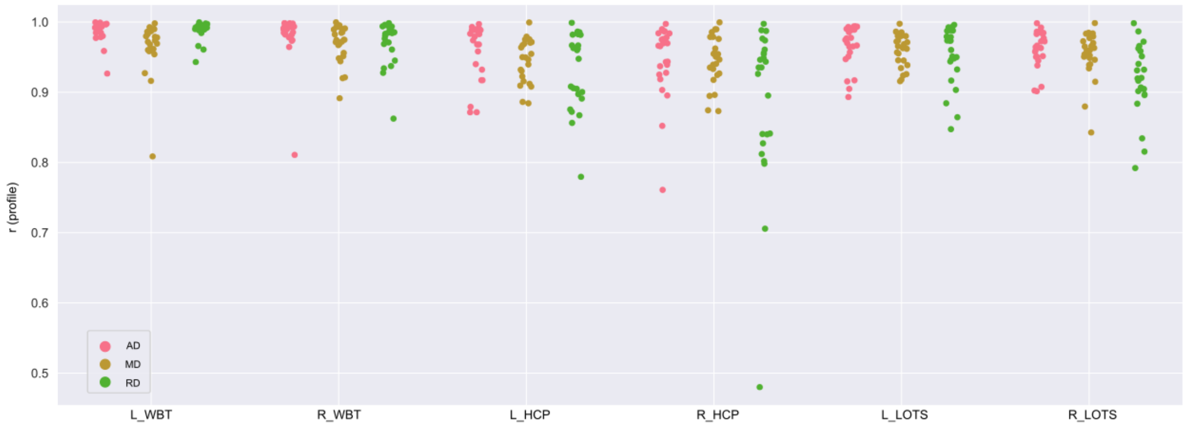

Figure S3. Test-retest reproducibility of fiber profiles for microstructural measurements
Correlation coefficients of fiber profiles on AD, MD and RD between test and retest computations. Each dot represents one instance of the correlation coefficient of one participant between test and retest computations.

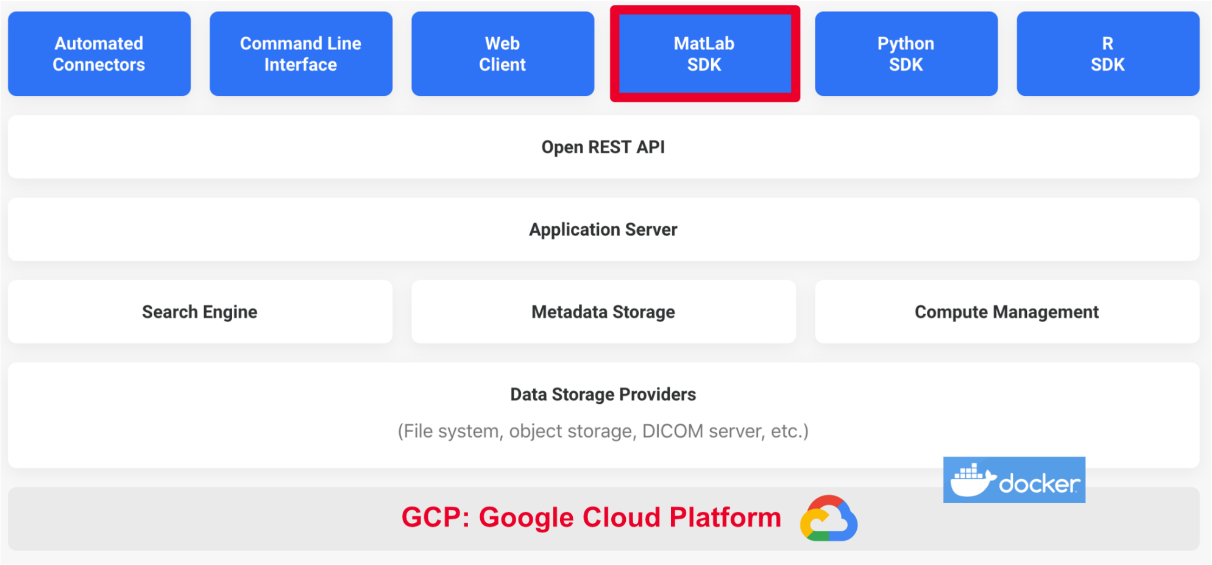

Figure S4. Flywheel's technical architecture

The first row (blue) shows the different ways to interact with the system: *(1) Automated connectors:* load data from the MRI scanners to Flywheel. *(2) Command Line Interface:* a program installed locally allows the authentication to the Flywheel system and most of the management operations. Useful, for example, to upload existing BIDS formated datasets to Flywheel if we don't have access to the original scanner. *(3) Web Client:* web interface to the Flywheel system accessible from all the major browsers. Allows the majority of the operations with a clean point and click interface. *(4-5-6) Matlab-Python-R SDK:* the SDK gives programmatic access to the Flywheel system, with the three main scientific programming languages. We marked Matlab SDK in red in Figure 5 because as part of the RTP2 solution we included Scitran (https://github.com/vistalab/scitran), which is a collection of Matlab scripts designed to interact programmatically with Flywheel. These interfaces use the Open (public) REST API (Representational State Transfer Application Programming Interface) to access Flywheel's Application Server, which control the web interface and Flywheel's three main systems: *(1) search engine:* accessible through the web GUI and from the SDK to search and retrieve only the required information, *(2) Metadata Storage:* core of the system, with references to all content, gear and analyses, and *(3) Compute management:* it manages the dispatching of jobs, i.e. it takes the input data, runs the Gear, and when finished, copies the results back to Flywheel. The last two rows represent the data storage and the actual hardware where Flywheel is running (and where the Gears run as well). In our specific instance of Flywheel, both the data and the computations happen in the Google Cloud Platform. Small Gears run on the same hardware as Flywheel, but big Gears (as all three gear composing RTP2) launch a new virtual machine each time, runs the Gear, and shuts it off when finished. There is a parameter in Flywheel that controls the number of machines that can run in parallel. This is required for both technical and economical reasons, as Google is not usually the bottleneck in this situation.
